# Assessing the associated medical, legal, and social issues in medical tourism and its implications for Nigeria

**DOI:** 10.11604/pamj.2023.45.145.41104

**Published:** 2023-07-31

**Authors:** Adeponle Olayode Adeoye

**Affiliations:** 1 Department of Sociology, Redeemer’s University, Ede, Osun State, Nigeria

**Keywords:** Health, medical tourism, government, treatment, Nigeria

## Abstract

Medical tourism is thriving in Nigeria among both elites and non-elites with over $1 billion annual expenditure on medical tourism. Inadequate healthcare infrastructure caused by economic problems, corruption and low budgetary allocation to the country’s health sector, and lack of trust in the country's healthcare systems to handle complex medical procedures have contributed to this trend. This article discusses the trend of medical tourism in Nigeria and the associated medical, legal, and social issues in medical tourism generally, relying on relevant literature. The paper concludes that medical tourism is not inherently dangerous; however, unequal economic and power status may influence the quality of hospitals where patients receive treatment and the quality of treatment received. This unequal power and economic status may also determine justice in cases of substandard treatment in foreign hospitals. The study recommends that the Nigerian government should improve healthcare systems in the country to reverse the trend of medical tourism and to reduce the financial burden that medical tourism exerts on average Nigerians who need a high level of care but cannot access it in their country. It is also recommended that a regulatory framework that ensures protection from substandard hospitals and justice for Nigerians who fall victim to substandard care abroad must be put in place by the Nigerian government. Relevant health stakeholders should also continue to sensitize the public about the complications that may be associated with some medical procedures sought outside Nigeria especially cosmetic surgery which may result in follow-up challenges.

## Essay

Medical tourism is a term used within the medical industry to describe the practice of seeking international medical services for emergency and non-emergency health situations [1]. Medical tourism is the practice of traveling from a country of residence to another country for the sole purpose of attaining healthcare services [2]. Medical tourism is an action taken to enhance or restore one´s health through medical services outside the jurisdiction of one´s national healthcare [3]. The definitions of medical tourism above imply that medical tourism involves crossing one´s national border to access medical services. The history of medical tourism can be traced to the 15^th^ century among Europeans when elites in Europe travel for spas and mineral baths in Mediterranean countries to improve their health [4]. In recent years, some countries have become world destinations for medical tourism like India, Thailand, Korea, Malaysia United Kingdom, the Middle East, Japan, the United States, Canada, Belgium, Costa Rica, Cuba, Dubai, Hungary, Israel, Jordan, Malaysia, Singapore, South Africa, and several others have also emerged as largest providers of international health services in the medical tourism business [5]. Many of these providers have grown exponentially to become a multi-billion-dollar industry and the largest receivers of medical tourists [5]. The worth of global medical tourism has been estimated to be 100 billion dollars and is projected to grow at an annual rate of 20% to 30% [6]. Popular healthcare services sought through medical tourism are; organ transplantation, reproductive treatment, and dental treatment [7]. The annual expenditure of Nigerians on international health services is estimated to be $1 billion with 60% of the money spent on oncology, orthopedics, nephrology, and cardiology [5]. This is also equivalent to government expenditure on the public health sector including salaries of the workers and government health intervention programs [5].

**Drivers of medical tourism in Nigeria:** the emerging breakthrough in medical technologies, the disparity in laws across countries concerning healthcare services, global economic inequities, and globalization promote medical tourism in the 21^st^ century world [8]. In Nigeria, the lingering economic situation has had a significant impact on the health sector in which the budgetary allocation to the sector is poor [9]. There is no doubt that most healthcare institutions in Nigeria underperform than that of the developed nations as they lack basic facilities and equipment expected of a healthcare institution [10]. Budgetary allocations and per capita government expenditure on health in Nigeria are far below what is required of a standard healthcare system [10]. This has incapacitated most healthcare institutions in terms of facilities and human resource development therefore contributing to a high rate of medical tourism among individuals who can afford such journeys. In low- and middle-income countries healthcare institutions like Nigeria, government hospitals lack basic diagnostic machines for chronic diseases and medical treatments for mild health conditions therefore patients die of unserious ailments such as diarrhea, malaria, injuries from accidents, tetanus, typhoid, and fever [10]. This has resulted in a lack of belief in Nigeria´s healthcare system among citizens of Nigeria. It should be noted that the practice of medical tourism is not only peculiar to the rich in Nigeria even individuals who are not able to afford such a journey go the extra mile in raising funds from social networks. Some others, even seek medical healthcare abroad when the health situation can be attended to in Nigeria because of the lack of belief in the Nigerian healthcare system [11]. A study conducted in Nigeria among individuals who have engaged in medical tourism and those who were about to go for medical tourism in Nigeria found that 40.2% strongly agree that improper medical treatment in the home country is the reason for their participation in outbound medical tourism [9]. It was also revealed that the majority of the respondents (47.7%) attributed infrastructural inadequacies as their reason for engaging in outbound medical tourism. More than half of the respondents (59.8%) claimed that they engage in outbound medical tourism because the treatment cost is commensurate to the quality of treatment abroad. 52.6% of the respondents were of very strong opinion that they engage in medical tourism because there are qualified health service providers abroad [9].

**Medical issues in medical tourism:** several ethical concerns have been raised about medical tourism. The practice of medical tourism has ethical implications at both individual and population health levels [12]. While some patients may receive good quality healthcare because of their economic strength and powerful status, some may suffer from medical complications after returning to their home country which might be a result of the low quality of care provided by foreign hospitals [12]. Some medical procedures carried out on patients might require follow-up to monitor the healing process [13]. However, because of the financial burden, most patients do not spend adequate time in foreign hospitals for proper monitoring of their health conditions. This usually becomes a burden to local doctors in medical tourists´ home countries as they bear the cost of correcting any negligent act caused by foreign medical providers [14]. For instance, a study conducted in a health facility in Nigeria among patients who presented themselves after receiving treatment abroad reported that 39% of patients who had neuro-surgical surgery died of complications upon returning to the country [15]. Over a quarter of these patients had infections that required follow-up care that was not initially planned [15]. Some of the health services that medical tourists seek may also be unethical and unlawful in their home country which may cause follow-up challenges upon completion of a procedure [16].

Medical tourists in the process of undergoing treatment abroad may be exposed to microbes that are not in existence or uncommon in their home country thereby facilitating the transmission of infectious diseases to their country of residence [2]. Such infectious diseases acquired abroad could lead to devastating outbreaks especially when the populace does not have existing inherent immunity to such diseases [2]. For instance, patients traveling for organ transplantation may be more susceptible to infectious complications as a result of poor screening protocols for organs in some foreign hospitals [17]. Cases where some patients acquired hepatitis B during cardiac surgery in Pakistan and renal transplantation in India have been documented [18]. The unethical practice of using foreign patients especially those from low- and middle-income countries for experimental treatment without informing them about the negative outcome associated with such procedure and the trend of organ harvesting of dead foreign patients without approval from the deceased family has been observed [19]. Curiosity about the efficacy of health professionals who carry out medical procedures on patients in foreign hospitals has also been raised. While several international private clinics offer services to tourists, some clinics may not be equivalent in terms of the standard stipulated by most medical regulatory bodies in regional countries [20]. Some of the clinics are certified as accredited facilities in collaboration with the government of the country to paint a picture that such clinics conduct health services according to globally established standards of quality and safety [20].

**Legal issues in medical tourism:** scholars have discussed the legal issues surrounding medical tourism. Medical tourists may be unable to seek legal justice for unsatisfactory treatment received in foreign countries, especially in countries where laws that protect tourist patients are not in existence [21]. Even in jurisdictions where such a law exists, it may be difficult to prove substandard care especially if the law relies heavily on evidence obtained from doctors in such countries [22]. International litigation is also expensive and difficult to enforce [21]. Therefore, patients who are not economically strong and unsatisfied with the medical services they received in foreign countries may be unable to seek justice. Traveling overseas to seek justice may exert another financial burden on the patients due to the cost of employing a suitable lawyer, travel expenses, and accommodation. Diverse laws that operate in different countries also make it difficult for patients to bring lawyers from their country of residence. Even when it is possible, language and cultural differences could also pose a challenge in seeking legal redress as foreign lawyers may not have the understanding of the courtroom if the jurisdiction for hearing the case is the country where the treatment was sought [23]. The most feasible option for such medical tourists who want justice is to employ a lawyer in the country where the medical procedure was done, which may also be expensive.

Even in cases where it is possible, a favorable judgment may not translate to enforcement of the judgment [23]. It is unlikely that such a judgment will be enforced, or they will get a financial reward. One of the reasons why United States health care is expensive is because of the legal right of patients to seek redress through judicial means in case of negligence or error while receiving care [23]. Also, some organ transplantation procedures carried out abroad are unlawful because they usually involve the purchase of organs. This often occurs in extremely poor societies where poverty clouds the thinking of the people about the consequences of selling their organs. A recent case is that of a former Nigerian lawmaker who has been convicted in the United Kingdom for coercing a young man into donating a kidney for a sick daughter through financial inducement without appropriately briefing the young man on the consequences of doing such [24].

**Social issues in medical tourism:** medical tourism is an act of neo-colonialism that makes low- and middle-income countries lack confidence in their healthcare system and dependent on the developed world [19]. Medical tourism reduces healthcare to a commodity, one that can only be accessed by privileged individuals. Medical tourism also contributes to the bad state of local healthcare systems in low-and middle-income countries as political leaders and other important stakeholders who should develop local healthcare systems have failed to do so due to medical tourism which provides them alternative healthcare access [7]. Medical tourism may exacerbate poverty and economic inequities in low- and middle-income countries as patients may exhaust their finances while trying to seek care abroad [25]. Such individuals may also lack the funds to manage complications that arise from such trips upon their return to their home country. As long as the option for treatment exists elsewhere, LMICs may feel little pressure to address the inadequacies in their healthcare system pushing citizens to risk material well-being in the pursuit of needed care [25]. Medical tourism may also lead to the substitution of clinical factors for financial reasons among patients due to the poor state of their local healthcare systems and the desperation to seek treatment. For instance, medical tourists may prefer to go to cheaper foreign hospitals than expensive ones without considering the standard of care in such hospitals [26].

**Implications of medical, legal, and social issues in medical tourism for Nigeria:** medical tourism is growing rapidly in Nigeria. However, unequal economic and power status in society may translate to differences in the quality of hospitals where patients go to receive treatments and the quality of treatments patients receive. The unequal power and economic status in society may also influence issues of justice when patients receive substandard care in foreign hospitals. Therefore, given the medical, legal, and social issues associated with medical tourism, Nigeria must first put in place machinery that would ensure that their healthcare systems are up to standards, and with state-of-the-art technologies to measure and meet up to the medical challenges of the citizens. This will drastically reduce the movement of patients from one country to another seeking healthcare service and reduce the financial burden that foreign medical trips put on average Nigerian families.

Since it is near impossible for a country to have all it takes for its citizens not to seek medical care abroad due to disparities in healthcare technologies and health systems efficiency across the world, Nigeria´s government must put enforceable legislations, policies, laws, and guidelines on medical tourism that regulates the quality and standard of hospitals where patients can seek medical care in foreign countries and that also protect their legal right in case of complications from substandard care. For instance, there should be a legal implication form to be signed by the treating physician/healthcare provider in the event of complications, or death of a patient caused by substandard care or if found to exploit organs of dead patients without appropriate consent from the patient´s family. Similarly, procedures of organ transplantation abroad should be made to pass through relevant governmental agencies to investigate who is donating and if appropriate inform consent has been done before such foreign trip is approved to proceed to foreign mission embassies to get visas. Relevant health stakeholders should also continue to sensitize the public about the complications that may be associated with some medical procedures sought outside Nigeria especially cosmetic surgery which may result in follow-up challenges when they come back to the country.

## Conclusion

Medical tourism is not a dangerous practice. However, the medical, legal, and social issues associated with medical tourism necessitate the need for the Nigerian government to reposition its healthcare systems to meet up to standards to reverse the trend of foreign medical travels and the complications that might be associated with this practice as well as the financial burden it exerts on average citizens who need care but cannot access it in their country. The associated legal issues also point to the fact that the government needs to regulate medical tourism to prevent Nigerians from falling into the hands of substandard foreign hospitals and to protect their right to timely justice in cases of substandard treatment. The public also needs to be reoriented about unethical cosmetic surgeries sought abroad which might result in complications upon their return to the country.
